# Editorial: Defining and Characterizing Respiratory Disease in an Aging Population

**DOI:** 10.3389/fmed.2022.889834

**Published:** 2022-04-07

**Authors:** Stefanie Krick, Patrick Geraghty, Claude Jourdan Le Saux, Mauricio Rojas, Claudia A. Staab-Weijnitz

**Affiliations:** ^1^Division of Pulmonary, Allergy and Critical Care Medicine, University of Alabama at Birmingham, Birmingham, AL, United States; ^2^SUNY Downstate Health Sciences University, Brooklyn, NY, United States; ^3^School of Medicine, University of California, San Francisco, San Francisco, CA, United States; ^4^Division of Pulmonary, Allergy and Critical Care, Department of Medicine, University of Pittsburgh, Pittsburgh, PA, United States; ^5^Institute of Lung Health and Immunity and Comprehensive Pneumology Center With the CPC-M BioArchive, Helmholtz Center München, Helmholtz Association of German Research Centres (HZ), Munich, Germany

**Keywords:** lung disease, aging, chronic obstructive pulmonary disease (COPD), idiopathic pulmonary fibrosis (IPF), pneumonia, cell senescence

Pulmonary function is one of the most compelling predictors of longevity (1). Major aging-associated chronic lung diseases including lung cancer, chronic obstructive pulmonary disease (COPD), and idiopathic pulmonary fibrosis (IPF) are currently lacking potently effective disease-modifying therapies, which makes geroscience highly applicable for finding novel anti-aging strategies in respiratory diseases. Furthermore, aging is an independent prediction factor for acute respiratory disease, especially in critically ill elderly patients (2, 3). This special issue topic invited contributions to not only showcase characteristics of aging-related respiratory diseases but also depict the underlying and potentially targetable molecular mechanisms. This issue accumulated 11 articles, including five original research articles, four reviews, one systematic review, and one opinion from a total of 90 authors from three different continents and six countries, which fit in to the following categories.

## Aging-Related Chronic Lung Disease Outcomes

Three of the featured studies are clinical studies including a prospective European multicenter cohort study, a retrospective cohort study using a database that included 18 registries, and a single-center prospective observational study.

The longitudinal German-wide INSIGHTS-IPF (Investigating major health trends in idiopathic pulmonary fibrosis) registry by Leuschner et al. specifically addressed IPF in advanced age. A significant portion of these IPF patients were older than 75 years and presented with several comorbidities and markedly decreased scores for quality of life. Nevertheless, they benefitted similarly from antifibrotic therapy as their younger IPF counterparts.

You et al. focused their retrospective study on a cohort of Chinese elderly patients (n = 53,694 patient cases), diagnosed with lung adenocarcinoma, which makes up 76% of lung cancer survivors. This patient group tends to exhibit worse tolerance to surgical and nonsurgical treatment modalities. You et al. generated a normogram including 11 prognostic factors, which proved to be reliable for determining the prognosis in elderly lung cancer patients.

Witt et al. identified handgrip strength as a component of the physical frailty phenotype in patients with COPD and an acute exacerbation, which is more common in the elderly patient, to correlate with increased 30-day readmission rate in this single US academic center study. Future studies will help to risk-stratify these patients and ultimately help improve their mortality and quality of life.

Taking a different perspective, Sucre et al. outline in a mini-review what is currently known about lung aging following neonatal chronic lung disease, i.e., bronchopulmonary dysplasia. In these patients, perinatal care including ventilation of the lung, albeit a life-saving measure, changes the future trajectory of lung development and aging in adult life. The authors describe underlying mechanisms and highlight the urgent need for profound exchange between healthcare professionals from pediatrics, primary care, and adult pulmonology.

## Acute Respiratory Diseases and Management in the Elderly Patient

In a mini-review on the impact of aging in acute respiratory distress syndrome (ARDS), Brown et al. outline the clinical evidence of implications of age in ARDS, and potential aging-associated mechanisms, including cellular senescence.

Two of the featured studies in our topic characterize acute respiratory disease and management of chronic respiratory failure in the elderly population. In a cross-sectional observational study from Switzerland/France, the investigators focused exclusively on individuals using non-invasive ventilation either at home or in a long-term facility showing that one-third were older than 75 years and this intervention was feasible. This warrants future studies to assess whether “out of hospital” non-invasive ventilation will also lead to improved quality of life in the elderly.

Chen et al. conducted a systematic meta-analysis, including a total of 17 retrospective studies encompassing 5,729 patients over 60 years old with pneumonia, to assess the distribution of pathogenic bacteria. They found a high prevalence of gram-negative bacilli (56%; most commonly *Klebsiella pneumonia* and *Pseudomonas aeruginosa*) and a 25% prevalence of gram-positive cocci (*Staphylococcus aureus* and Streptococcus species). This meta-analysis can help guide antibiotic treatment choices not only for Chinese elderly patients but also globally.

## Mechanisms of Lung Aging in Health and Disease

The two main molecular and cellular mechanisms in this special issue are age-associated accumulation of senescent cells and alterations in extracellular matrix (ECM) components.

Sullivan et al. set out to establish a human alveolar cell culture model of telomere-induced senescence based on the lung adenocarcinoma cell line A549, characterizing its transcriptome and secretome. In contrast to murine data, they found robust transcriptional induction of senescence-associated secretory phenotype genes as well as senescence-associated changes in the secretome, suggesting species-specific responses to telomere dysfunction identified four novel senescence-associated peripheral biomarkers associated with IPF.

The opinion article by Meiners and Lehmann focuses on reparative senescence during lung fibrosis and highlights recent observations that challenge the traditional understanding of senescence.

Non-coding RNAs, including long non-coding RNAs and micro-RNAs, are key regulators of senescence and emerge as targetable biomolecules for the treatment of many diseases, including COPD and IPF therapy. Omote and Sauler summarize the current literature about the contributions of non-coding RNAs in IPF, COPD, and lung aging. They highlight the potential and the challenges of targeting non-coding RNAs for the treatment of chronic lung disease in the elderly.

ECM composition is altered during lung aging. The comprehensive review on changes in collagen biosynthesis, processing, and maturation in the aging lung by Onursal et al. harnesses recent observations on the expression of collagens, the collagen biosynthetic machinery, as well as collagen degradation pathways in the aging lung.

In summary, our Special Issue showcases diversity in clinical and basic aging research focusing on lung disease, which we hope will help stimulate further research to address a timely and relevant worldwide problem with respiratory diseases being in the top five death-causing ailments and a growing aging global population.

## Author Contributions

SK and CS-W drafted the manuscript. All authors contributed to writing this Editorial of the Research Topic on characterizing respiratory disease in an aging population that they edited in 2019.

## Funding

SK acknowledges support from the National Institutes of Health (NIH, R01HL160911). PG is supported by DOD PR190071 and the Alpha-1 Foundation 614218. CJ is supported by NIH (U01HL134766 and R35HL150767). CS-W acknowledges support from the Friedrich-Baur-Stiftung (Reg.-Nr. 51/16), the Deutsche Forschungsgemeinschaft (DFG) within the Research Training Group GRK2338, the Helmholtz Association, and the German Center for Lung Research (DZL).

## Conflict of Interest

The authors declare that the research was conducted in the absence of any commercial or financial relationships that could be construed as a potential conflict of interest.

## Publisher's Note

All claims expressed in this article are solely those of the authors and do not necessarily represent those of their affiliated organizations, or those of the publisher, the editors and the reviewers. Any product that may be evaluated in this article, or claim that may be made by its manufacturer, is not guaranteed or endorsed by the publisher.
